# Experiential learning in interprofessional health training on chronic non-communicable diseases: an *ex-post-facto* evaluation

**DOI:** 10.1590/1980-220X-REEUSP-2024-0300en

**Published:** 2025-05-09

**Authors:** Saulo Fabio Ramos, Felipa Rafaela Amadigi, Natália Gonçalves, Thaise Honorato de Souza, Vânia Marli Schubert Backes, Francis Solange Vieira Tourinho, Betina Hörner Schlindwein Meirelles, Monica Motta Lino

**Affiliations:** 1 Universidade Federal de Santa Catarina, Programa de Pós-Graduação de Enfermagem, Florianópolis, SC, Brazil.

**Keywords:** Education, Public Health Professional, Chronic Disease, Patient Care Bundles, Interprofessional Education, Primary Health Care

## Abstract

**Objective:**

To characterize Intervention Projects in light of Kolb’s Experiential Learning Theory, focusing on training health professionals to manage chronic non-communicable diseases.

**Method:**

Ex-post facto research with analysis of 337 Intervention Projects from the Specialization Course on Care for People with Chronic Diseases, provided by the Universidade Federal de Santa Catarina (2022–2023). The analysis applied the Experiential Learning Theory, with an emphasis on integrated and interprofessional interventions.

**Results:**

The projects addressed diabetes, hypertension, obesity, and smoking, with interventions aimed at education, promotion, and management of chronic conditions using multidisciplinary approaches. The capillarization of training allowed the adaptation of interventions to regional needs, promoting collaborative practices among different health professionals. Kolb’s Theory has proven effective in transforming care and management practices, especially in regions with distinct socioeconomic challenges.

**Conclusion:**

Kolb’s Theory has proven effective in interprofessional training and in the development of practical skills for the management of chronic non-communicable conditions, promoting integrated care adapted to local specificities.

## INTRODUCTION

The Strategic Action Plan to Tackle Chronic Diseases and Non-Communicable Injuries (*Dant*), created in 2021, has, among its recommendations, the training of health professionals capable of working in an integrated manner, with effective communication, and presentation of positive results in health education and empowerment of the population to manage chronic diseases. The complexity of the healthcare field requires highly qualified professionals to deal with constantly changing clinical and social challenges. Therefore, their training must be effective and aligned with current clinical practice^(1)^, as is the case in the context of chronic non-communicable diseases (CNCD).

Chronic Health Conditions (CHC) education promotes collaboration among physicians, nurses, nutritionists, physical therapists, and other professionals to provide comprehensive care to people. Continuing education in health is based on meaningful learning and the possibility of transformation, generating reflection on the work process, self-management, institutional change, and transformation of practices in service. Interprofessional education has been gaining prominence in recent years due to its ability to improve the quality of health care, improve the qualifications of professionals, and provide more appropriate training for students in the health field^(2,3)^.

Among the skills identified as essential, effective communication has gained prominence. To communicate effectively, professionals require training that ensures them the knowledge and skills necessary to achieve this objective, and that gives them the competence to do so. Having the ability to establish communication focused on the population’s needs is one of them. Evidence shows that patient-centered communication involving healthcare professionals, individuals, and caregivers increases satisfaction and better adherence to treatment, and can achieve health outcomes that are very close to ideal^(4,5)^. In the context of people with chronic conditions, this competence can be applied in terms of chronic care management, such as in Obesity, Systemic Arterial Hypertension (SAH), and Diabetes Mellitus (DM).

The inclusion of a health education intervention on these diseases, their relationships, risk factors, and modifiable habits using the ability to dialogue with the population involved, focusing on the greatest needs and/or knowledge deficits, without the professionals seeming to show their knowledge on the subject, allowing the population to share with them their experience with chronic conditions, has proven to be effective in improving knowledge, verbal expression about the understanding of the disease, as well as its probable consequences and the possibility of choosing a healthy lifestyle^(6,7)^.

In this context, this article presents an ex-post-facto impact assessment of the Specialization Course on Care for People with Chronic Non-Communicable Diseases. The Course was provided by Universidade Federal de Santa Catarina (UFSC) in 2022 and 2023 and is part of the macroproject entitled “Professional and Managerial Qualification of Santa Catarina professionals in Chronic Non-Communicable Diseases” – a proposal developed by an interdisciplinary team of researchers, professors, and health managers from the State of Santa Catarina, Brazil.

In the field of education, it is important to address and improve concepts and definitions with the aim of improving the skills and competencies necessary for managing professional activities. Care management becomes complex when it combines management concepts and health practices with cognitive, analytical, and behavioral skills to effectively integrate the whole to promote effective performance in the provision of care.

Implementing changes in health and care services is often hindered by organizational micropolicies. There are calls for those leading change to develop and use policy skills and behaviors to understand and mediate these policies, but to date only limited research has offered a developed empirical conceptualization of policy skills and behaviors for leading change in health services^(8)^.

The study aimed to analyze Intervention Projects (IP) from the perspective of Experiential Learning Theory (ELT), which defends the individual’s ability to construct and reconstruct their knowledge based on lived experiences^(8)^. Originally developed in the field of education, this approach has been widely applied in various professional training contexts. Individuals who have this orientation generally make decisions intuitively, are capable of change, and value interpersonal relationships.

The ELT, which finds wide application in the educational sector, provides a significant theoretical framework for the study in question. This theory proposes that learning is created from the modification of lived experiences, which stimulates reflections and causes substantial changes in professional practices. Therefore, when examining Intervention Projects in light of ELT, we seek to deepen the understanding of the opportunities and challenges of continuing education in the context of CNCD care management. This approach aims to fill existing gaps in the literature by integrating aspects of management, professional training, and educational innovation.

## METHOD

*Ex-post facto* Impact Assessment Research, using cause-effect assessment^(9)^, that is, the study was carried out after an intervention – in this case, the Specialization Course on Care for People with Chronic Diseases, provided by Universidade Federal de Santa Catarina (2022–2023) with the adoption of Kolb’s Experiential Learning Theory. Most health professionals who finished the course were nurses, doctors, pharmacists, physiotherapists, nutritionists, dentists, among others, who demonstrated the theoretical-methodological characteristics and results of professional training. Based on ELT, the period of data collection and analysis took place from August 2023 to August 2024, after the end of the specialization course, being the product of a doctoral dissertation in nursing and having 337 IPs as data source.

The organization of the IPs was led by a team of trained researchers. The documents were inserted in the software Atlas Ti®. The texts were coded in light of the Theory of Experiential Learning considering a) sociodemographic characterization of health professionals and subsequent categorization according to sex, age, education, municipality of origin, areas of activity; b) theme of the intervention project giving rise to the categories: Diabetes Mellitus, Obesity, Systemic Arterial Hypertension and Smoking c) type of proposed intervention that gave rise to the following categories: Health Education, Promotion, and Management.

The analysis of the 337 IPs was carried out using the principles of Kolb’s experiential learning theory and followed a theoretical saturation strategy. During the data coding and categorization process, new themes were continually analyzed until no new information emerged that expanded the existing categories. This procedure ensured that the data analyzed were sufficient to meet the study objectives and reflect the variety of interventions performed.

The information related to the characterization of the graduates underwent simple descriptive analysis, while the remaining was subjected to pre-content analysis and exploration of the material, followed by coding and categorization of the data, then to the treatment of the results, which included inference and interpretation considering Prodanov and Freitas’ recommendations^(9)^ for qualitative research^(10)^.

The study follows the recommendations of the Consolidated Criteria for Reporting Qualitative research (COREQ)^(11)^. It was forwarded to and approved by the Ethics Committee with number CAAE 66635322.0.0000.0121, opinion number 6.078.402. Its development followed the standards of Resolution No. 466 of 2012, of the National Health Council, and the General Data Protection Law (LGPD) which establishes the rules for handling personal data. The authors report no conflicts of interest in the development of this research. The 337 IPs were coded to preserve the authors’ anonymity and received the sequential identification from IP 1 to IP 337.

## RESULTS

The characterization of graduates revealed a female predominance (84.87%), with nurses being the majority (58.16%), followed by nutritionists (13.65%), pharmacists (7.12%), dentists (4.45%), doctors (4.15%), physiotherapists (3.86%), and other health professionals (8.61%) (Table 1). The distribution of professionals by hubs showed that the capital, Florianópolis, had the highest representation with 8 graduates (24.92%), followed by the cities of Blumenau with 69 (20.47%), Joinville with 61 (18.10%), Criciúma with 54 (16.04%), Chapecó with 48 (14.24%), and Lages with 21 (6.23%). The intervention themes were organized into four domains of the Experiential Learning Theory: Identification, Analysis, Problematization, and Transformation. The most frequently addressed conditions included Diabetes Mellitus (36.40%), Systemic Arterial Hypertension (22.90%), Obesity (15.80%), and Smoking (9.10%). The most frequently performed interventions were health education (37.43%), health promotion (32.30%), and health management (15.21%). Florianópolis stood out as the hub with the highest number of interventions on the topics of Diabetes Mellitus (45%) and Hypertension (32%).

**Table 1:** Distribution of graduates by sex, profession and intervention themes – Florianópolis, SC, Brazil, 2024. (n = 337).

Variable	Frequency (%)	Hubs n (%)					
**Main themes**		**Blumenau**	**Chapecó**	**Criciuma**	**Florianopolis**	**Joinville**	**Lages**
Diabetes mellitus Arterial Hypertension Obesity Others* Smoking	164 (36.40) 103 (22.90) 71 (15.80) 71 (15.80) 41 (9.10)	26 (39.4) 15 (22.7) 9 (13.6) 11 (16.7) 5 (7.6)	28 (42.4) 13 (19.7) 10 (15.2) 10 (15.2) 5 (7.6)	25 (41) 12 (19.7) 11 (18) 7 (11.5) 6 (9.8)	45 (33.3) 32 (23.7) 19 (14.1) 25 (18.5) 14 (10.4)	30 (34.5) 20 (23) 17 (19.5) 14 (16.1) 6 (6.9)	10 (28.6) 11 (31.4) 5 (14.3) 4 (11.4) 5 (14.3)
**Main interventions**		**Blumenau**	**Chapecó**	**Criciuma**	**Florianopolis**	**Joinville**	**Lages**
Health education Health promotion Health management Technological interventions Situational diagnosis Others**	241 (37.43) 208 (32.30) 98 (15.21) 48 (7.45) 46 (7.14)	31 (33) 26 (27.7) 22 (23.4) 9 (9.6) 6 (6.4) 0	36 (38.3) 29 (30.9) 11 (11.7) 7 (7.4) 10 (10.6) 1 (1.1)	35 (36.5) 30 (31.3) 17 (17.7) 4 (4.2) 9 (9.4) 1 (1.1)	69 (34.7) 69 (34.7) 29 (14.6) 14 (7) 18 (9) 0	49 (43.4) 37 (32.7) 16 (14.2) 9 (8) 2 (1.8) 0	21 (43.8) 17 (35.4) 3 (6.3) 5 (10.4) 1 (2.1) 1 (2.1)

* Renal, mental, neurological, respiratory, cardiovascular, endocrine, and neoplastic conditions; treatment.

** Work process, professional identity, Brazilian Public Health system (*SUS*) pharmacy.

Regarding the interventions carried out by graduates, 37.43% were focused on health education, especially aimed at people with Diabetes Mellitus and Hypertension, with a strong participation of nurses and nutritionists. Interventions aimed at Health promotion represented 32.30%, with greater participation of physiotherapists and pharmacists, mainly in smoking cessation and obesity control initiatives. Interventions related to health management accounted for 15.21% of the projects, where nurses, doctors, and dentists played a crucial role in planning and monitoring the interventions.

An important highlight was the interprofessional nature of 31% of the interventions, which involved teams made up of different health professionals (nurses, nutritionists, doctors, physiotherapists, among others), aiming at more integrated and effective care. These interprofessional interventions were particularly notable in health promotion and education, where collaboration among different professional categories resulted in multidisciplinary approaches to the management of chronic conditions such as obesity and smoking.

By hub, the most common interventions carried out in Florianópolis were focused on the themes of Diabetes Mellitus (45%) and Hypertension (32%), with deep involvement of nurses and doctors. Blumenau stood out in health promotion initiatives, especially in obesity control, with greater participation of nutritionists and physiotherapists. Joinville presented a balanced distribution between education and health promotion, with pharmacists playing an important role in smoking cessation actions (10%).

In Criciúma and Chapecó, most interventions focused on health education, with a predominance of nurses and nutritionists in awareness-raising activities on hypertension and diabetes. In Lages, where the representation of professionals was smaller (6.23%), the actions were more directed towards the management of chronic conditions, with dentists and physiotherapists leading oral health monitoring programs and physical activities to control hypertension.

## DISCUSSION

The Specialization Course on Care for People with CNCD, provided by UFSC, brought significant contributions to the qualification of professionals, evidencing the capillarization of the course in several regions of the State of Santa Catarina. The female predominance among graduates reflects the reality of the health workforce, with women playing a fundamental role in the care of CNCD. This leading role is especially notable among nurses, who lead the consolidation of Primary Health Care (PHC), identifying community needs and empowering people to care for their health conditions^(11,12,13)^. Historically, Nursing is a predominantly female profession, while areas such as Medicine, with greater social prestige, were occupied by men^(11)^. Nursing professionals demonstrate strong alignment with ELT, using their daily practice to address issues and reflect on applied knowledge, strengthening their role as leaders in CNCD care^(14)^.

The distribution of graduates across the hubs of Florianópolis, Lages, Blumenau, Joinville, Chapecó, and Criciúma reinforces the scope of the course. Diabetes (36.40%), followed by Hypertension (22.90%), Obesity (15.80%), and Smoking (9.10%) were the most prevalent conditions addressed by the intervention projects, reflecting the urgent need for educational and health promotion strategies. Between 2006 and 2020, the obesity trend increased by 9.7 pp, going from 11.8% in 2006 to 21.5% in 2020. During the period analyzed, the prevalence of arterial hypertension and diabetes mellitus among adults living in Brazilian capitals showed relative stability, with values around 25.2% and 8.2%, respectively. The prevalence of diabetes and hypertension is higher among less educated people, while those with more education exhibit healthier behaviors^(15)^. Another survey confirms that the proportion of people with obesity continued to increase between 2022 and 2023, rising from 21.7% to 22.8%^(16)^.

The analysis of the projects revealed broad training, qualifying professionals to deal with chronic conditions in different territories. The *DANT* plan is aligned with national indicators and targets, has the potential to significantly impact the reduction of morbidity and mortality due to CNCD and strengthen intersectoral and networked work processes, promoting innovation and new professional skills for health promotion^(17)^. Data reinforce the global trend of increasing non-communicable chronic conditions, which require urgent prevention and control actions. In 2019, 54.7% of deaths in Brazil were attributed to CNCD, with emphasis on cardiovascular diseases, cancers, diabetes, and chronic respiratory diseases. These problems are closely linked to living conditions and unequal access to public goods and services, such as education, employment, and health^(18)^.

The State of Santa Catarina recorded an increase in its premature mortality rates due to CNCD in the period studied and did not reach the targets agreed for the period^(19)^. Population coverage by PHC teams decreased between 2017 and 2018 in the State, and this fact was not associated with the increase in the premature mortality rate due to CNCD. Despite the drop in premature mortality rates due to CNCD in Brazil and efforts in Primary Care, control of this mortality is still unstable^(19)^. In addition to CNCD prevention and control policies, it is crucial to analyze the factors that influence this premature mortality^(19)^.

The topics covered by the graduates were broad, approaching areas such as mental health, neoplasms, lifestyle, health management, integrated care, alternative therapies, specific population health, drug management and interventions, professional aspects and identity. These themes highlight the scope of the course and the complexity of CNCDs. Markers of chronic morbidities, self-care, risk factors and user profile, together with adherence to services and a multidisciplinary approach, are key strategies for training and health planning^(20)^.

Interventions implemented by graduates, based on the Theory of Experiential Learning^(10)^, included health education, health promotion, management of chronic conditions, and multidisciplinary approaches. These interventions not only align with the principles of the theory based on a continuous four-stage cycle (concrete experience (CE) – Act; reflective observation (RO) – Reflect; abstract conceptualization (AC) – Conceptualize; active experimentation (EA) - Apply^(8)^), but also meet specific needs of users, providing an integrated and collaborative approach to the care of chronic conditions. Health education has proven to be a transformative tool, especially on topics such as diabetes, hypertension, obesity and smoking, providing practical information for self-care and behavior change^(21,22)^. Community activities, such as walks and support groups for smoking cessation, reinforce the importance of health promotion in creating environments that encourage healthy lifestyles^(22)^.

Interventions guided by health education are essential so that people can manage their chronic conditions more effectively. Diabetes, hypertension, obesity, and smoking education programs aim to provide practical and relevant information to enable users to better understand their disease and adopt self-care behaviors. These interventions reflect the experiential learning cycle^(15,23)^, in which specific experiences and thoughts are used to promote lasting changes in behavior^(10,23)^.

Community activities that promote healthy lifestyles, such as walking, recreational areas, healthy eating groups are examples of strategies that promote public health. These activities provide opportunities for physical activity, and also create the supportive environment needed for behavior change. Health Promotion involves strategies that consider both individual aspects, promoting healthier lifestyles, and collective aspects, recognizing the conditions that facilitate or hinder the adoption of these options^(22)^. From a positive perspective, it focuses more on health than illness. Understanding the salutogenic approach has driven innovation in theory and design of health interventions that differentiate actions with medical approaches and care systems^(24)^.

Care for chronic conditions requires an individualized approach, which requires an individual treatment plan that meets the specific needs of each user, with interventions such as regular blood pressure monitoring, diet monitoring, blood glucose monitoring, and obesity monitoring. Kolb’s theory^(11,15)^ emphasizes the importance of adapting learning strategies to individual needs, which is reflected in the development and implementation of these programs. Each interaction with a patient is an opportunity to learn more about their needs and adjust treatment plans accordingly, promoting a continuous cycle of learning and development, such as Individualized Treatment Plans. ELT establishes the learning process for managers, teams and organizations, for problem-solving, decision-making, seeking opportunities for entrepreneurship and for formulating strategies^(18)^. Thus, managerial administrators tend to be directed to initiate programs that promote “Experiential Learning” and find assumptions for their current work^(11,18,23)^.

Managing chronic conditions requires an individualized and ongoing approach, focusing on interventions such as blood pressure monitoring, blood glucose control, and nutrition. ELT emphasizes adapting learning strategies to patients’ needs, promoting the development of individualized treatment plans^(22)^. Interprofessionality emerges as an essential factor in the care of CNCD, with the collaboration of several health professionals, such as nutritionists, physiotherapists, psychologists and doctors, being fundamental for the implementation of more comprehensive and effective strategies^(22)^.

The training provided by UFSC, which integrated several health professions, served as a practical example of the application of experiential learning to develop interprofessional skills essential to the management of CNCD. This approach facilitated understanding and respect for the roles of each professional within the team, promoting more coordinated and integrated care^(15,22)^.

To justify ELT-based interventions carried out by trained health professionals, focusing on chronic conditions, it is essential to understand how these actions are in line with the principles of this theory. According to Kolb, learning occurs through the transformation of experience, in a cyclical process that combines concrete experiences, reflections, abstractions, and experiments^(8)^. This approach proves particularly effective in the management of chronic conditions, as it allows health professionals to adjust their strategies to the specific needs of users, promoting significant changes in care practices.

Graduates of the Specialization Course, in turn, developed projects in six hubs in Santa Catarina, categorized into four main types of interventions: Health Education, Health Promotion, and Management of Chronic Conditions. Each of these initiatives benefited from ELT, integrating theoretical knowledge and reflective practice. This way, professionals were able to not only address risk factors associated with chronic conditions — as evidenced in national studies that reflect the increase in obesity and the prevalence of hypertension and type 2 diabetes^(11,12)^, but also to improve the quality of care and promote actions that meet regional peculiarities.

This approach allowed the identification of relevant areas for investigation, development of more effective public guidelines, and implementation of actions that meet local needs. ELT, therefore, provided a solid theoretical framework for the ongoing training of professionals and for improving public health in Santa Catarina.

The use of Kolb’s Experiential Learning Theory^(15,23)^ allowed a practical and reflective approach for the professionals involved. Continuing education, based on real and significant experiences, promoted the transformation of care and management practices, highlighting the importance of continuous training and meaningful learning for the management of CNCD^(25,26)^. This approach is aligned with the National Plan to tackle CNCDs 2021–2030, and denotes the importance of continuing education to prevent risk factors and promote the health of the population^(18)^. Verifying health data is essential for evidence-based decision-making, promoting improved health system management and the involvement of people receiving care^(27)^.

The training of workers from different professional groups promotes comprehensive and cooperative action in the care of Chronic Conditions. Kolb’s theory^(11,15)^ emphasizes the importance of experience-based learning in interdisciplinary collaboration, in which the exchange of knowledge and experiences among specialists promotes more effective management of chronic diseases. This type of interaction facilitates understanding the complexity of each condition and the creation of more comprehensive and appropriate treatment strategies for patients, reinforcing the importance of a multidisciplinary and integrated approach.

Interprofessional education in health strengthens the collaborative logic in health work, seeking to reduce the duplication of professional acts, improve communication, increase users’ satisfaction and ensure patient safety through the integration of actions^(22)^. Interprofessionality promotes collaborative work among health professionals, strengthening relationships within universities, services and communities, in addition to developing critical professionals and better meeting the needs of the community^(22)^.

Interprofessional engagement and collaborative practice are essential for quality public health and the contribution to primary health care. Particularly, given the increasing prevalence of non-communicable diseases, core competencies in professional teamwork are essential for preparing the health and social workforces of the 21st century^(28,29)^.

The findings of this research highlight the importance of incorporating interprofessional practices and continuing education in the management of Noncommunicable Chronic Conditions. The implementation of Kolb’s ELT provided a practical and reflective basis, facilitating the evolution of the participants’ care and management practices. Interprofessionality, which is the focus of the interventions examined, emerges as an important strategy in improving the quality of care and addressing the complexities of CNCD management, taking into account socioeconomic and cultural aspects.

The difference in interventions based on ELT lies in their experiential nature, which allows professionals to apply theoretical knowledge in practical contexts, reflecting on the results to optimize future approaches. This continuous learning cycle was particularly effective in developing critical skills and adapting strategies to local needs and to other regions covered by the course.

Increasingly, interprofessional teamwork is necessary for the effective provision of public health services in primary health care settings, in line with the principles of the *SUS*. Interprofessional competencies should therefore be incorporated into all health and social service education programs. Educational innovation in the development of student-led clinics (SLC) provides a unique opportunity to assess and develop such competencies^(30)^. Finally, the capillarization of training stands out as a key factor, with the distribution of trained professionals in different regions of Santa Catarina, allowing interventions to be adapted to local needs.

Experiential learning, by valuing direct experience and critical reflection, ensures that professionals are prepared to face regional challenges and promote more equitable and effective public health^(8,22,23)^. It is also worth highlighting the merit of frequently evaluating the teamwork climate in health services, with a view to providing support for promoting actions that aim to improve this aspect of interprofessional work and, consequently, promote a work environment that is more favorable to interprofessional collaboration.

### Limitations and Indications for Future Studies

Although data were widely explored, the non-use of all available tools in the software AtlasTi® stands out as a possible limitation. Future studies may explore the adaptation of the Experiential Learning Theory in other states of the country.

## CONCLUSION

The intervention projects developed throughout the Specialization Course on Care for People with Chronic Non-Communicable Diseases demonstrated a significant impact, contributing to interprofessional training and practice, to expand skills in the management of chronic conditions. The application of the Theory of Experiential Learning stood out as a strategy to integrate reflective practice into the real context, promoting transformations in care and management approaches. The interventions carried out addressed conditions such as diabetes, hypertension, obesity, and smoking, aligning with local epidemiological needs and reinforcing the role of continuing education in building solutions adapted to regional demands. The interprofessional nature of the interventions, in addition to promoting collaboration between different professional categories, demonstrated potential to improve care coordination and enhance the quality of life of the communities served.

These results indicate that the combination of specialized training, experiential learning, and interprofessional strategies has the potential to contribute to strengthening the management of care for chronic non-communicable conditions, aligning health practices with contemporary social and epidemiological demands. The study emphasizes the relevance of educational models integrating theory and practice as pillars for continuous and meaningful training, with a direct impact on the quality of health care.
