# Supplementary material for: Experiential learning in interprofessional health training on chronic non-communicable diseases: an ex-post-facto evaluation
Source: Rev Esc Enferm USP. 2025 May 9;59:e20240300. doi: 10.1590/1980-220X-REEUSP-2024-0300en (PMC12068242; doi:10.1590/1980-220X-REEUSP-2024-0300en)
Supplement: Translation

# Translation: Aprendizagem experiencial na formação interprofissional em saúde sobre condições crônicas não transmissíveis: uma avaliação *ex-post-facto*

**DOI:** 10.1590/1980-220X-REEUSP-2024-0300pt

**Authors:** Saulo Fabio Ramos, Felipa Rafaela Amadigi, Natália Gonçalves, Thaise Honorato de Souza, Vânia Marli Schubert Backes, Francis Solange Vieira Tourinho, Betina Hörner Schlindwein Meirelles, Monica Motta Lino

**Affiliations:** 1 Universidade Federal de Santa Catarina, Programa de Pós-Graduação de Enfermagem, Florianópolis, SC, Brasil.

**Keywords:** Educação Profissional em Saúde Pública, Doenças crônicas, Pacotes de Assistência ao Paciente, Educação Interprofissional, Atenção Primária à Saúde

## Abstract

**Objetivo:**

Caracterizar Projetos de Intervenção à luz da Teoria da Aprendizagem Experiencial de Kolb, focando na capacitação em profissionais de saúde para o manejo de condições crônicas não transmissíveis.

**Método:**

Pesquisa ex-post facto com análise de 337 Projetos de Intervenção do Curso de Especialização em Atenção às Pessoas com Condições Crônicas, oferecido pela Universidade Federal de Santa Catarina (2022–2023). A análise aplicou a Teoria da Aprendizagem Experiencial, com ênfase em intervenções integradas e interprofissionais.

**Resultados:**

Os projetos abordaram diabetes, hipertensão, obesidade e tabagismo, com intervenções direcionadas à educação, promoção e gestãoem condições crônicas utilizandoabordagens multidisciplinares. A capilarização da formação permitiu adaptar as intervenções às necessidades regionais, promovendo práticas colaborativas entre diferentes profissionais de saúde. A Teoria de Kolb mostrou-se eficaz na transformação das práticas assistenciais e gerenciais, especialmente em regiões com desafios socioeconômicos distintos.

**Conclusão:**

A Teoria de Kolb mostrou-se eficaz na formação interprofissional e no desenvolvimento de competências práticas para o manejo de condições crônicas não transmissíveis, promovendo cuidados integrados e adaptados às especificidades locais.

## INTRODUÇÃO

O Plano de Ações Estratégicas para o Enfrentamento das Doenças Crônicas e Agravos Não Transmissíveis (Dant), criado em 2021, tem entre suas recomendações a formação de profissionais de saúde capazes de trabalhar de forma integrada, com comunicação efetiva e apresentação de resultados positivos na educação em saúde e empoderamento da população para o manejo das condições crônicas. A complexidade da área da saúde exige profissionais altamente qualificados para lidar com os desafios clínicos e sociais em constante mudança. Assim, sua formação deve ser eficaz e alinhada à prática clínica atual^(1)^, como é o caso do contexto das condições crônicas não transmissíveis (CCNT).

A educação em Condições Crônicas de Saúde (CCS) promove a colaboração entre médicos, enfermeiros, nutricionistas, fisioterapeutas e outros profissionais para oferecer um cuidado integral às pessoas. A educação permanente em saúde baseia-se na aprendizagem significativa e na possibilidade de transformação, gerando reflexão do processo de trabalho, autogestão, mudança institucional e transformação das práticas em serviço. A educação interprofissional vem se destacando ao longo dos últimos anos em razão de sua capacidade de ampliar a qualidade da atenção à saúde, melhorar a qualificação dos profissionais e a formação mais adequada dos estudantes da área da saúde^(2,3)^.

Dentre as competências identificadas como essenciais, a comunicação efetiva tem ganhado destaque. Para comunicar-se efetivamente, o profissional precisa ter uma formação que lhe garanta os conhecimentos ehabilidades necessárias para alcançá-la e que lhe confiraa competência para tal. Ter a aptidão de estabelecer uma comunicação centrada nas necessidades da população é uma delas. Evidências mostram que a comunicação centrada no paciente envolvendo os profissionais de saúde, pessoas e cuidadores aumenta a satisfação e a melhor adesão ao tratamento, podendo alcançar resultados de saúde bem próximos aos ideais^(4,5)^. No contexto de pessoas com condições crônicas esta competênciapode ser aplicada, em termos de gestão de cuidados crônicos, como na Obesidade, Hipertensão Arterial Sistêmica (HAS) e Diabetes Mellitus (DM).

A inclusão de uma intervenção de educação em saúde sobre estas doenças, suas relações, fatores de risco e hábitos modificáveis utilizando-se da habilidade de dialogar com a população envolvida, focando nas maiores necessidades e/ou déficits de conhecimento, sem queos profissionais pareçam mostrar o seu conhecimento sobre o assunto, possibilitando à população compartilhar com eles a sua experiência com as condições crônicas, mostra ser eficaz para a melhora do conhecimento, da expressão verbal sobre a compreensão da doença, bem como das suas prováveis consequências e a possibilidade de escolha de um estilo de vida saudável^(6,7)^.

Neste contexto, o presente artigo apresenta uma avaliação de impacto do tipo *ex-post-facto* do Curso de Especialização em Atenção às Pessoas com Doenças Crônicas Não-Transmissíveis. O Curso foi ofertado pela Universidade Federal de Santa Catarina (UFSC) nos anos de 2022 e 2023 e integra o macroprojeto intitulado “Qualificação Profissional e de Gestores de Santa Catarina em Doenças Crônicas Não-Transmissíveis” – uma proposta elaborada em equipe interdisciplinar de pesquisadores, professores e gestores em saúde do Estado de Santa Catarina, Brasil.

No domínio da educação, é importante abordar e melhorar conceitos e definições com o objetivo de melhorar as aptidões e competências necessárias à gestão das atividades profissionais. A gestão do cuidado torna-se complexa quando combina conceitos de gestão e práticas em saúde com habilidades cognitivas, analíticas e comportamentais para integrar efetivamente o todo para promover um desempenho eficaz na prestação de cuidados.

A implementação de mudanças nos serviços de saúde e de cuidados é, muitas vezes, complicada por micropolíticas organizacionais. Há apelos para que aqueles que lideram a mudança desenvolvam e utilizem competências e comportamentos políticos para compreender e mediar essas políticas, mas até a data apenas uma investigação limitada oferece uma conceptualização empírica desenvolvida das competências e comportamentos políticos para liderar a mudança nos serviços de saúde(8).

O estudo teve como propósito analisar os Projetos de Intervenção (PI) sob a perspectiva da Teoria da Aprendizagem Experiencial (TAE), que defende a capacidade do indivíduo de construir e reconstruir seu conhecimento a partir das experiências vivenciadas^(8)^. Originalmente desenvolvida no campo da educação, essa abordagem tem sido amplamente aplicada em diversos contextos de formação profissional. Os indivíduos que possuem esta orientação geralmente tomam decisões intuitivamente, são aptos a mudanças e valorizam os relacionamentos interpessoais.

A TAE, que encontra ampla aplicação no setor educacional, ofereceum referencial teórico significativo para o estudo em questão. Essa teoria propõe que o aprendizado é criado a partir da modificação das experiências vividas, que estimula reflexões e provoca mudanças substanciais nas práticas profissionais. Portanto, ao examinar os Projetos de Intervenção à luz da TAE, procura-se aprofundar a compreensão das oportunidades e desafios da educação contínua no contexto da gestão do cuidado das CCNT. Essa abordagem visa suprir lacunas existentes na literatura ao integrar aspectosde gestão, formação profissional e inovação educacional.

## MÉTODO

Pesquisa de Avaliação de Impacto do tipo *ex-post facto*, de avaliação causa-efeito^(9)^, ou seja, o estudo foi realizado após uma intervenção - no caso, o Curso de Especialização em Atenção às Pessoas com Condições Crônicas, oferecido pela Universidade Federal de Santa Catarina (2022–2023) com adoção da Teoria da Aprendizagem Experiencial de Kolb. A maioria dos profissionais da saúde egressos era composta de enfermeiros, médicos, farmacêuticos, fisioterapeutas, nutricionista, cirurgião dentistas, entre outros, que mostraram as características teórico-metodológicas e os resultados de formação profissional. Pautado na TAE, o período da coleta e análise dos dados transcorreu de agosto de 2023 a agosto de 2024, após o término do curso de especialização, sendo produto de uma tese de doutorado em enfermagem e tendo como fonte de dados 337 Projetos de Intervenção (PIs).

A organização dos PIs foi conduzida por equipe de pesquisadores treinados. Os documentos foram inseridos no *software* Atlas Ti®. Os textos foram codificados à luz da Teoria da Aprendizagem Experiencial considerando a) caracterização sociodemográfica dos profissionais de saúde e, posteriormente, categorização segundo sexo, idade, formação, município de origem, polos de atuação. b) tema do projeto de intervenção dando origem às categorias: Diabetes Mellitus, Obesidade, Hipertensão Arterial Sistêmica e Tabagismo c) tipo de intervenção proposta que deu origem as seguintes categorias: Educação, Promoção e Gestão em Saúde.

A análise dos 337 projetos de intervenção foi realizada utilizando os princípios da teoria da aprendizagem experiencial de Kolb e seguiu uma estratégia de saturação teórica. Durante o processo de codificação e categorização dos dados, novos temas foram continuamente analisados até que não surgissem novas informações que ampliassem as categorias existentes. Este procedimento garantiu que os dados analisados fossem suficientes para atender aos objetivos do estudo e refletir a variedade de intervenções realizadas.

As informações relacionadas à caracterização dos egressos foram submetidas à análise descritiva simples, enquanto as demais foram submetidas à pré-análise de conteúdo e exploração do material, seguida de codificação e categorização dos dados, ascendendo ao tratamento dos resultados, que incluiu inferência e interpretação considerando as recomendações Prodanov e Freitas^(9)^ para pesquisas qualitativas^(10)^.

O estudo atende às recomendações do *Consolidated Criteria for Reporting Qualitative Research* (COREQ). A pesquisa foi encaminhada e aprovada no Comitê de Ética com número CAAE 66635322.0.0000.0121, número do parecer 6.078.402. O seu desenvolvimento seguiu as normas da Resolução nº 466 de 2012, do Conselho Nacional de Saúde e a Lei Geral de Proteção de Dados (LGPD) que institui as normas com as diligências com os dados pessoais. Os autores informam inexistir conflitos de interesses no desenvolvimento da presente pesquisa. Os 337 PIs foram codificados para preservar o anonimato dos autores e receberam a identificação sequencial de PI 1 até PI 337.

## RESULTADOS

A caracterização dos egressos revelou uma predominância feminina (84,87%), com enfermeiras sendo a maioria (58,16%), seguidas por nutricionistas (13,65%), farmacêuticos (7,12%), cirurgiões-dentistas (4,45%), médicos (4,15%), fisioterapeutas (3,86%) e outros profissionais de saúde (8,61%) (Tabela 1). A distribuição dos profissionais por polos mostrou que a capital, Florianópolis, teve a maior representatividade com 8 egressos (24,92%), seguida por Blumenau com 69 (20,47%), Joinville com 61 (18,10%), Criciúma com 54 (16,04%), Chapecó com 48 (14,24%) e Lages com 21 (6,23%). Os temas de intervenção foram organizados em quatro domínios da Teoria da Aprendizagem Experiencial: Identificação, Análise, Problematização e Transformação. As condições mais abordadas incluíram Diabetes Mellitus (36,40%), Hipertensão Arterial Sistêmica (22,90%), Obesidade (15,80%) e Tabagismo (9,10%). As intervenções mais realizadas foram educação em saúde (37,43%), promoção da saúde (32,30%) e gestão em saúde (15,21%). Florianópolis destacou-se como o polo com maior número de intervenções nos temas de Diabetes Mellitus (45%) e Hipertensão (32%).

**Tabela 1:** Distribuição dos egressos por sexo, profissão e temas de intervenção - Florianópolis, SC, Brasil, 2024. (n = 337).

Variável	Frequência (%)	Polos n (%)					
**Temas principais**		**Blumenau**	**Chapecó**	**Criciúma**	**Florianópolis**	**Joinville**	**Lages**
Diabetes Mellitus Hipertensão Arterial Obesidade Outros* Tabagismo	164 (36,40) 103 (22,90) 71 (15,80) 71 (15,80) 41 (9,10)	26 (39,4) 15 (22,7) 9 (13,6) 11 (16,7) 5 (7,6)	28 (42,4) 13 (19,7) 10 (15,2) 10 (15,2) 5 (7,6)	25 (41) 12 (19,7) 11 (18) 7 (11,5) 6 (9,8)	45 (33,3) 32 (23,7) 19 (14,1) 25 (18,5) 14 (10,4)	30 (34,5) 20 (23) 17 (19,5) 14 (16,1) 6 (6,9)	10 (28,6) 11 (31,4) 5 (14,3) 4 (11,4) 5 (14,3)
**Principais intervenções**		**Blumenau**	**Chapecó**	**Criciúma**	**Florianópolis**	**Joinville**	**Lages**
Educação em saúde Promoção da saúde Gestão em saúde Intervenções tecnológicas Diagnóstico situacional Outros**	241 (37,43) 208 (32,30) 98 (15,21) 48 (7,45) 46 (7,14) 3 (0,47)	31 (33) 26 (27,7) 22 (23,4) 9 (9,6) 6 (6,4) 0	36 (38,3) 29 (30,9) 11 (11,7) 7 (7,4) 10 (10,6) 1 (1,1)	35 (36,5) 30 (31,3) 17 (17,7) 4 (4,2) 9 (9,4) 1 (1,1)	69 (34,7) 69 (34,7) 29 (14,6) 14 (7) 18 (9) 0	49 (43,4) 37 (32,7) 16 (14,2) 9 (8) 2 (1,8) 0	21 (43,8) 17 (35,4) 3 (6,3) 5 (10,4) 1 (2,1) 1 (2,1)

* Condições renais, mentais, neurológicas, respiratórias, cardiovasculares, endócrinas e neoplasias; tratamento.

** Processo de trabalho, identidade profissional, farmácia do SUS.

No que diz respeito às intervenções realizadas pelos egressos, 37,43% foram focadas em educação em saúde, especialmente direcionadas às pessoas com Diabetes Mellitus e Hipertensão, com uma forte participação de enfermeiros e nutricionistas. As intervenções direcionadas à Promoção da saúde representaram 32,30%, com maior participação de fisioterapeutas e farmacêuticos, principalmente em iniciativas de cessação do tabagismo e controle da obesidade. Já as intervenções relacionadas à gestão em saúde foram responsáveis por 15,21% dos projetos, onde enfermeiras, médicos e cirurgiões-dentistas desempenharam um papel crucial no planejamento e monitoramento das intervenções.

Um destaque importante foi o caráter interprofissional de 31% das intervenções, que envolveram equipes compostas por diferentes profissionais da saúde (enfermeiros, nutricionistas, médicos, fisioterapeutas, entre outros), visando um cuidado mais integrado e eficaz. Essas intervenções interprofissionais foram particularmente notáveis na promoção e educação em saúde, nas quais a colaboração entre as diferentes categorias profissionais resultou em abordagens multidisciplinares para o manejo de condições crônicas, como a obesidade e o tabagismo.

Por polo, as intervenções mais realizadas em Florianópolis concentraram-se nas temáticas Diabetes Mellitus (45%) e Hipertensão (32%), com forte atuação de enfermeiros e médicos. Blumenau destacou-se em iniciativas de promoção da saúde, especialmente no controle da obesidade, com maior participação de nutricionistas e fisioterapeutas. Joinville apresentou uma distribuição equilibrada entre educação e promoção da saúde, com farmacêuticos desempenhando um papel importante em ações de cessação do tabagismo (10%).

Em Criciúma e Chapecó, a maioria das intervenções focou em educação em saúde, com uma predominância de enfermeiros e nutricionistas nas atividades de conscientização sobre hipertensão e diabetes. Já em Lages, onde a representatividade de profissionais foi menor (6,23%), as ações foram mais direcionadas para a gestão de condições crônicas, com cirurgiões-dentistas e fisioterapeutas liderando programas de monitoramento de saúde bucal e atividades físicas para controle da hipertensão.

## DISCUSSÃO

O Curso de Especialização em Atenção às Pessoas com CCNT, oferecido pela UFSC, trouxe contribuições significativas à qualificação dos profissionais, evidenciando a capilarização no curso por diversas regiões do Estado de Santa Catarina. A predominância feminina entre os egressos reflete a realidade da força de trabalho em saúde, com mulheres desempenhando um papel fundamental no cuidado das CCNT. Esse protagonismo é especialmente notável entre as enfermeiras, que lideram a consolidação na Atenção Primária à Saúde (APS), identificando necessidades comunitárias e empoderando pessoas no cuidado de suas condições de saúde^(11–13)^. Historicamente, Enfermagem é uma profissão majoritariamente feminina, enquanto áreas como Medicina, de maior prestígio social, eram ocupadas por homens^(11)^. Os profissionais de Enfermagem demonstram um forte alinhamento com a TAE, utilizando sua prática diária para resolver questões e refletir sobre o conhecimento aplicado, fortalecendo seu papel como líderes no cuidado de CCNT^(14)^.

A distribuição dos egressos pelos polos de Florianópolis, Lages, Blumenau, Joinville, Chapecó e Criciúma reforça o alcance do curso. O Diabetes (36,40%), seguido pela Hipertensão (22,90%), Obesidade (15,80%) e Tabagismo (9,10%) foram as condições mais prevalentes abordadas pelos projetos de intervenção, refletindo a necessidade urgente de estratégias educativas e de promoção da saúde. Entre 2006 e 2020, a tendência de obesidade aumentou 9,7 p.p., saindo de 11,8% em 2006 para 21,5% em 2020. Durante o período analisado, as prevalências de hipertensão arterial e diabetes mellitus entre adultos residentes nas capitais brasileiras apresentaram relativa estabilidade, com valores em torno de 25,2% e 8,2%, respectivamente. A prevalência de diabetes e hipertensão é maior entre as pessoas menos escolarizadas, ao mesmo tempo em que as mais escolarizadas apresentam comportamentos mais saudáveis^(15)^. Outra pesquisa confirma que a proporção de pessoas com obesidade continuou aumentando entre 2022 e 2023, saindo de 21,7% para 22,8%^(16)^.

A análise dos projetos revelou uma formação ampla, capacitando profissionais para lidar com as condições crônicas nos diversos territórios. O plano DANT está alinhado aos indicadores e metas nacionais, têm o potencial de impactar significativamente a redução da morbimortalidade por CCNT e fortalecer processos de trabalho intersetoriais e em rede, promovendo inovação e novas competências profissionais para a promoção da saúde^(17)^. Os dados reforçam a tendência global de aumento das condições crônicas não transmissíveis, que exigem ações urgentes de prevenção e controle. Em 2019, 54,7% dos óbitos no Brasil foram atribuídos às CCNT, com destaque para as doenças cardiovasculares, cânceres, diabetes e doenças respiratórias crônicas. Esses agravos estão intimamente ligados às condições de vida e ao acesso desigual a bens e serviços públicos, como educação, emprego e saúde^(18)^.

O Estado de Santa Catarina registrou aumento de suas taxas de mortalidade prematura por CCNT no período estudado e não atingiu as metas pactuadas para o período^(19)^. A cobertura populacional pelas equipes de APS diminuiu entre os anos de 2017 e 2018 no Estado, e este fato não teve associação com o aumento da taxa de mortalidade prematura por CCNT. Apesar da queda nas taxas de mortalidade prematura por CCNT no Brasil e dos esforços na Atenção Básica, o controle dessa mortalidade ainda é instável^(19)^. Além das políticas de prevenção e controle das CCNT, é crucial analisar os fatores que influenciam essa mortalidade prematura^(19)^.

Os temas abordados pelos egressos foram amplos, cobrindo áreas como saúde mental, neoplasias, estilo de vida, gestão em saúde, cuidados integrados, terapias alternativas, saúde específica da população, gestão e intervenções de medicamentos, aspectos profissionais e identidade. Esses temas evidenciam a abrangência do curso e a complexidade das CCNT. Marcadores de morbidades crônicas, autocuidado, fatores de risco e perfil dos usuários, junto à adesão aos serviços e uma abordagem multidisciplinar, são estratégias-chave para capacitação e planejamento em saúde^(20)^.

As intervenções implementadas pelos egressos, baseadas na Teoria da Aprendizagem Experiencial^(10)^, incluíram educação em saúde, promoção da saúde, gestão de condições crônicas e abordagens multidisciplinares. Essas intervenções não apenas se alinham aos princípios da teoria com base em um ciclo contínuo de quatro estágios (a experiência concreta (EC) – Agir; observação reflexiva (RO) – Refletir; conceituação abstrata (CA) – Conceitualizar; experimentação ativa (EA) – Aplicar^(8)^), mas também atendem às necessidades específicas dos usuários, fornecendo uma abordagem integrada e colaborativa no cuidado às condições crônicas. A educação em saúde demonstrou ser uma ferramenta transformadora, especialmente em temas como diabetes, hipertensão, obesidade e tabagismo, fornecendo informações práticas para o autocuidado e a mudança de comportamento^(21,22)^. As atividades comunitárias, como caminhadas e apoio de grupos à cessação do tabagismo, reforçam a importância da promoção da saúde na criação de ambientes que incentivam estilos de vida saudáveis^(22)^.

As intervenções pautadas pela educação em saúde são imprescindíveis para que as pessoas possam gerir as suas condições crônicas de forma mais eficaz. Os programas de educação sobre diabetes, hipertensão, obesidade e tabagismo visam fornecer informações práticas e relevantes para permitir que os usuários compreendam melhor sua doença e adotem comportamentos de autocuidado. Estas intervenções refletem o ciclo de aprendizagem experiencial^(15,23)^, em que experiências e pensamentos específicos são usados para promover mudanças duradouras de comportamento^(10,23)^.

As atividades comunitárias que promovem estilos de vida saudáveis, como caminhadas, áreas de lazer, grupos de alimentação saudável são exemplos de estratégias que promovem a saúde pública. Essas atividades proporcionam oportunidades para atividade física, e ainda criam o ambiente de apoio necessário para a mudança de comportamento. A Promoção da Saúde envolve estratégias que consideram tanto os aspectos individuais, promovendo modos de vida mais saudáveis, quanto os coletivos, ao reconhecer as condições que facilitam ou dificultam a adoção dessas opções^(22)^. Numa perspectiva positiva, concentra-se mais na saúde do que na doença. O entendimento da abordagem salutogênica tem impulsionado inovação na teoria e na concepção de intervenções em saúde que diferenciam as ações com enfoques médicos e dos sistemas de atenção^(24)^.

A atenção às condições crônicas exige uma abordagem individualizada, o que exige um plano de tratamento individual que atenda às necessidades específicas de cada usuário, com intervenções como monitoramento regular da pressão arterial, monitoramento da dieta, monitoramento da glicemia e da obesidade. A teoria de Kolb^(11,15)^ enfatiza a importância da adaptação das estratégias de aprendizagem às necessidades individuais, o que se reflete no desenvolvimento e implementação destes programas. Cada interação com um paciente é uma oportunidade para aprender mais sobre suas necessidades e ajustar os planos de tratamento de acordo, promovendo um ciclo contínuo de aprendizado e desenvolvimento, como os Planos de Tratamento Individualizados. A TAE estabelece o processo de aprendizagem pelos gestores, equipes e organizações, para resolução de problemas, tomada de decisão, busca de oportunidades para empreender e para formulação de estratégias^(18)^. Desta forma, os gestores gerenciais tendem a ser guiados a iniciar programas que promovam a “Aprendizagem Experiencial” e encontrem pressupostos para o seu trabalho atual^(11,18,23)^.

A gestão de condições crônicas exige uma abordagem individualizada e contínua, com foco em intervenções como o monitoramento da pressão arterial, controle da glicemia e nutrição. A TAE enfatiza a adaptação das estratégias de aprendizagem às necessidades dos pacientes, promovendo o desenvolvimento de planos de tratamento individualizados^(22)^. A interprofissionalidade emerge como um fator essencial no cuidado das CCNT, com a colaboração de diversos profissionais de saúde, como nutricionistas, fisioterapeutas, psicólogos e médicos, sendo fundamental para a implementação de estratégias mais abrangentes e eficazes^(22)^.

A formação oferecida pela UFSC, que integrou diversas profissões da saúde, serviu como um exemplo prático da aplicação da aprendizagem experiencial para desenvolver competências interprofissionais essenciais ao manejo das CCNT. Essa abordagem facilitou a compreensão e o respeito pelos papéis de cada profissional dentro da equipe, promovendo cuidados mais coordenados e integrados^(15,22)^.

Para justificar as intervenções baseadas na TAE, realizadas por profissionais de saúde formados, com foco nas condições crônicas, é fundamental entender como essas ações estão em consonância com os princípios dessa teoria. Segundo Kolb, o aprendizado acontece por meio da transformação da experiência, em um processo cíclico que combina vivências concretas, reflexões, abstrações e experimentações^(8)^. Essa abordagem revela-se particularmente eficaz no manejo de condições crônicas, pois permite que os profissionais de saúde ajustem suas estratégias às necessidades específicas dos usuários, promovendo mudanças significativas nas práticas de cuidado.

Os egressos do Curso de Especialização, por sua vez, desenvolveram projetos em seis polos de Santa Catarina, categorizados em quatro tipos principais de intervenções: Educação em Saúde, Promoção da Saúde e Gestão de Condições Crônicas. Cada uma dessas iniciativas beneficiou-se da TAE, integrando conhecimento teórico e prática reflexiva. Dessa maneira, os profissionais puderam não apenas abordar fatores de risco associados às condições crônicas — como evidenciado em estudos nacionais que refletem o aumento da obesidade e a prevalência de hipertensão e diabetes tipo 2^(11,12)^, mas também elevar a qualidade do cuidado e promover ações que atendem às peculiaridades regionais.

Essa abordagem possibilitou a identificação de áreas relevantes para investigação, desenvolver diretrizes públicas mais eficazes e aplicar ações que atendam às necessidades locais. A TAE, dessa forma, ofereceu um referencial teórico sólido para a capacitação contínua dos profissionais e para a melhoria da saúde pública em Santa Catarina.

A utilização da Teoria da Aprendizagem Experiencial de Kolb^(15,23)^ permitiu uma abordagem prática e reflexiva para os profissionais envolvidos. A educação permanente, baseada em experiências reais e significativas, promoveu a transformação das práticas assistenciais e gerenciais, destacando a importância da formação contínua e da aprendizagem significativa para o manejo das CCNT^(25,26)^. Essa abordagem está alinhada ao Plano Nacional de Enfrentamento das CCNT 2021–2030, denota a importância da educação permanente para prevenir fatores de risco e promover a saúde da população^(18)^. A verificação de dados em saúde é essencial para a tomada de decisões baseadas em evidências, promovendo a melhoria da gestão do sistema de saúde e o envolvimento das pessoas cuidadas^(27)^.

A formação de trabalhadores dos diversos núcleos profissionais promove uma atuação integral e cooperativa nos cuidados das Condições Crônicas. A teoria de Kolb^(11,15)^ enfatiza a importância da aprendizagem baseada na experiência na colaboração interdisciplinar, em que a troca de conhecimentos e experiências entre especialistas promove uma gestão mais eficaz das doenças crônicas. Este tipo de interação facilita a compreensão da complexidade de cada agravo e a criação de estratégias de tratamento mais abrangentes e adequadas aos pacientes, reforçando a importância de uma abordagem multidisciplinar e integrada.

A educação interprofissional em saúde fortalece a lógica colaborativa no trabalho em saúde, buscando reduzir a duplicação de atos profissionais, aprimorar a comunicação, aumentar a satisfação dos usuários e garantir a segurança do paciente por meio da integração das ações^(22)^. A interprofissionalidade promove o trabalho colaborativo entre profissionais de saúde, fortalecendo relações entre universidade, serviços e comunidades, além de desenvolver profissionais críticos e atender melhor às necessidades da comunidade^(22)^.

O envolvimento interprofissional e a prática colaborativa são essenciais para a saúde pública de qualidade e a contribuição nos cuidados à saúde primária. Especialmente dada a crescente prevalência de doenças não transmissíveis, as competências fundamentais do trabalho em equipe profissional são essenciais para a preparação das forças de trabalho sociais e de saúde do século^(28,29)^.

Os achados desta pesquisa ressaltam a importância de incorporar práticas interprofissionais e educação continuada no manejo das Condições Crônicas Não Transmissíveis. A implementação da TAE de Kolb ofereceu uma base prática e reflexiva, facilitando a evolução das práticas assistenciais e gerenciais dos participantes. A interprofissionalidade, que é o foco das intervenções examinadas, emerge como uma estratégia importante na melhoria da qualidade do cuidado e enfrentamento às complexidades no manejo das CCNT, levando em conta aspectos socioeconômicos e culturais.

O diferencial das intervenções baseadas na TAE reside em seu caráter experiencial, que possibilita aos profissionais aplicar conhecimentos teóricos em contextos práticos, refletindo sobre os resultados para otimizar futuras abordagens. Esse ciclo contínuo de aprendizado foi particularmente eficaz no desenvolvimento de competências críticas e na adaptação de estratégias às necessidades locais e às de outras regiões abrangidas pelo curso.

Cada vez mais, o trabalho em equipe interprofissional é necessário para a prestação eficaz de serviços de saúde pública em ambientes de atenção primária à saúde, articulado com os princípios do SUS. Competências interprofissionais devem, portanto, ser incorporadas em todos os programas de educação em saúde e serviço social. A inovação educacional no desenvolvimento de clínicas lideradas por estudantes (SLC) oferece uma oportunidade única para avaliar e desenvolver tais competências^(30)^. Por fim, a capilarização da formação destaca-se como um fator-chave, com a distribuição dos profissionais formados em diferentes regiões de Santa Catarina, permitindo que as intervenções fossem adaptadas às necessidades locais.

A aprendizagem experiencial, ao valorizar a experiência direta e a reflexão crítica, garante que os profissionais estejam preparados para enfrentar desafios regionais e promover uma saúde pública mais equitativa e eficaz^(8,22,23)^. Ressalta-se também o mérito de avaliar frequentemente o clima de trabalho em equipe nos serviços de saúde, visando fornecer subsídios para promover ações que visem melhorar esse aspecto do trabalho interprofissional e, consequentemente, promover um ambiente de trabalho mais favorável à colaboração interprofissional.

### Limitações e Indicações para Estudos Futuros

Embora os dados tenham sido amplamente explorados, destaca-se como uma possível limitação a não utilização de todas as ferramentas disponíveis no *software* AtlasTi®. Futuros estudos podem explorar a adaptação da Teoria da Aprendizagem Experiencial em outros Estados do país.

## CONCLUSÃO

Os projetos de intervenção desenvolvidos ao longo do Curso de Especialização em Atenção às Pessoas com Condições Crônicas Não Transmissíveis demonstraram um impacto significativo, contribuindo para a formação e prática interprofissional, para ampliação de competências no manejo das condições crônicas. A aplicação da Teoria da Aprendizagem Experiencial destacou-se como uma estratégia para integrar a prática reflexiva ao contexto real, promovendo transformações nas abordagens assistenciais e gerenciais. As intervenções realizadas abordaram condições como diabetes, hipertensão, obesidade e tabagismo, alinhando-se às necessidades epidemiológicas locais e reforçando o papel da educação permanente na construção de soluções adaptadas às demandas regionais. O caráter interprofissional das intervenções, além de promover colaboração entre diferentes categorias profissionais, demonstrou potencial para melhorar a coordenação do cuidado e ampliar a qualidade de vida das comunidades atendidas.

Esses resultados indicam que a combinação entre formação especializada, aprendizagem experiencial e estratégias interprofissionais tem potencial para contribuir para o fortalecimento da gestão do cuidado das condições crônicas não transmissíveis, alinhando as práticas de saúde às demandas sociais e epidemiológicas contemporâneas. O estudo reforça a relevância de modelos educativos que integrem teoria e prática como pilares para uma formação contínua e significativa, com impacto direto na qualidade da atenção à saúde.

# Translation: Aprendizaje experiencial en la formación interprofesional en salud sobre enfermedades crónicas no transmisibles: una evaluación *ex-post-facto*

**DOI:** 10.1590/1980-220X-REEUSP-2024-0300es

**Authors:** Saulo Fabio Ramos, Felipa Rafaela Amadigi, Natália Gonçalves, Thaise Honorato de Souza, Vânia Marli Schubert Backes, Francis Solange Vieira Tourinho, Betina Hörner Schlindwein Meirelles, Monica Motta Lino

**Affiliations:** 1 Universidade Federal de Santa Catarina, Programa de Pós-Graduação de Enfermagem, Florianópolis, SC, Brasil.

**Keywords:** Educación en Salud Pública Profesional, Enfermedad Crónica, Paquetes de Atención al Paciente, Educación Interprofesional, Atención Primaria de Salud

## Abstract

**Objetivo:**

Caracterizar Proyectos de Intervención a la luz de la Teoría del Aprendizaje Experiencial de Kolb, centrándose en la formación de profesionales de la salud para el manejo de enfermedades crónicas no transmisibles.

**Método:**

Investigación ex post facto con análisis de 337 Proyectos de Intervención del Curso de Especialización en Atención a Personas con Condiciones Crónicas, ofrecido por la Universidad Federal de Santa Catarina (2022–2023). El análisis aplicó la Teoría del Aprendizaje Experiencial, con énfasis en intervenciones integradas e interprofesionales.

**Resultados:**

Los proyectos abordaron la diabetes, la hipertensión, la obesidad y el tabaquismo, con intervenciones orientadas a la educación, la promoción y el manejo de enfermedades crónicas utilizando enfoques multidisciplinarios. La capilarización de la formación permitió adaptar las intervenciones a las necesidades regionales, promoviendo prácticas colaborativas entre diferentes profesionales de la salud. La teoría de Kolb ha demostrado ser eficaz para transformar las prácticas de atención y gestión, especialmente en regiones con distintos desafíos socioeconómicos.

**Conclusión:**

La Teoría de Kolb ha demostrado ser eficaz en la formación interprofesional y en el desarrollo de habilidades prácticas para el manejo de enfermedades crónicas no transmisibles, promoviendo una atención integrada adaptada a las especificidades locales.

## INTRODUCCIÓN

El Plan de Acción Estratégico para el Abordaje de las Enfermedades Crónicas y No Transmisibles, creado en 2021, tiene entre sus recomendaciones la formación de profesionales de la salud capaces de trabajar de forma integrada, con comunicación efectiva y presentación de resultados positivos en educación para la salud y empoderamiento de la población para el manejo de las condiciones crónicas. La complejidad en salud requiere profesionales altamente cualificados para hacer frente a los retos clínicos y sociales en constante evolución. Por ello, su formación debe ser eficaz y estar en consonancia con la práctica clínica actual^(1)^, como ocurre en el contexto de las enfermedades crónicas no transmisibles (ECNT). La Educación en Condiciones Crónicas de Salud (ECS) promueve la colaboración entre médicos, enfermeros, nutricionistas, fisioterapeutas y otros profesionales para ofrecer una atención integral a las personas. La educación sanitaria permanente se basa en el aprendizaje significativo y la posibilidad de transformación, generando reflexión sobre el proceso de trabajo, la autogestión, el cambio institucional y la transformación de las prácticas de servicio. La educación interprofesional ha cobrado protagonismo en los últimos años debido a su capacidad para aumentar la calidad de la atención sanitaria, mejorar la cualificación de los profesionales y proporcionar una formación más adecuada a los estudiantes sanitarios^(2,3)^.

Entre las competencias identificadas como esenciales, ha cobrado protagonismo la comunicación eficaz. Para comunicarse eficazmente, los profesionales necesitan tener una formación que les garantice los conocimientos y habilidades necesarios para lograrlo y les proporcione la competencia para hacerlo. Tener la capacidad de establecer una comunicación centrada en las necesidades de la población es una de ellas. Está demostrado que la comunicación centrada en el paciente, en la que participan los profesionales sanitarios, las personas y los cuidadores, aumenta la satisfacción y la adherencia al tratamiento, y puede lograr resultados de salud muy próximos a los ideales^(4,5)^. En el contexto de las personas con enfermedades crónicas, esta competencia puede aplicarse en términos de gestión de cuidados crónicos, como la Obesidad, la Hipertensión Arterial Sistémica (HAS) y la Diabetes Mellitus (DM).

La inclusión de una intervención de educación para la salud sobre estas enfermedades, sus relaciones, factores de riesgo y hábitos modificables utilizando la capacidad de dialogar con la población involucrada, centrándose en las mayores necesidades y/o déficits de conocimiento, sin que los profesionales aparezcan mostrando sus conocimientos sobre el tema, permitiendo que la población comparta con ellos su experiencia con las condiciones crónicas, demuestra ser eficaz para mejorar el conocimiento, la expresión verbal sobre la comprensión de la enfermedad, así como sus probables consecuencias y la posibilidad de elegir un estilo de vida saludable^(6,7)^.

En este contexto, este artículo presenta una evaluación de impacto *ex-post-facto* del Curso de Especialización en Atención a Personas con Enfermedades Crónicas No Transmisibles. El curso fue ofrecido por la Universidad Federal de Santa Catarina (UFSC) en 2022 y 2023 y forma parte del macroproyecto titulado «Cualificación de Profesionales y Gestores en Santa Catarina en Enfermedades Crónicas No Transmisibles» – una propuesta elaborada por un equipo interdisciplinario de investigadores, docentes y gestores en salud del estado de Santa Catarina, Brasil.

En el campo de la educación, es importante abordar y mejorar los conceptos y definiciones con el objetivo de mejorar las habilidades y competencias necesarias para gestionar las actividades profesionales. La gestión asistencial se vuelve compleja cuando combina conceptos de gestión y prácticas en salud con competencias cognitivas, analíticas y comportamentales para integrar eficazmente el conjunto y promover un desempeño eficaz en la prestación de cuidados.

La aplicación de cambios en los servicios sanitarios y asistenciales suele verse complicada por la micropolítica organizativa. Se pide a quienes lideran el cambio que desarrollen y utilicen habilidades y comportamientos políticos para comprender y mediar en estas políticas, pero hasta la fecha sólo unas pocas investigaciones ofrecen una conceptualización empírica desarrollada de las habilidades y comportamientos políticos para liderar el cambio en los servicios sanitarios^(8)^.

El propósito de este estudio fue analizar los Proyectos de Intervención (PI) desde la perspectiva de la Teoría del Aprendizaje Experiencial (TAE), que defiende la capacidad del individuo para construir y reconstruir su conocimiento a partir de sus experiencias^(8)^. Desarrollado originalmente en el ámbito de la educación, este enfoque se ha aplicado ampliamente en diversos contextos de formación profesional. Los individuos con esta orientación suelen tomar decisiones de forma intuitiva, son propensos al cambio y valoran las relaciones interpersonales.

El TAE, ampliamente aplicado en el sector educativo, ofrece un marco teórico significativo para el estudio que nos ocupa. Esta teoría propone que el aprendizaje se crea a partir de la modificación de las experiencias vividas, lo que estimula la reflexión y provoca cambios sustanciales en las prácticas profesionales. Por lo tanto, al examinar los Proyectos de Intervención a la luz de la TAE, pretendemos profundizar en nuestra comprensión de las oportunidades y los retos de la formación continua en el contexto de la gestión de los cuidados ECNT. Este enfoque pretende llenar lagunas en la literatura integrando aspectos de gestión, formación profesional e innovación educativa.

## MÉTODO

Se trató de un estudio de evaluación de impacto *ex-post facto*, causa-efecto^(9)^, es decir, el estudio se realizó después de una intervención – en este caso, el Curso de Especialización en Atención a Personas con Condiciones Crónicas, ofrecido por la Universidad Federal de Santa Catarina (2022–2023), adoptando la Teoría del Aprendizaje Experiencial de Kolb. La mayoría de los profesionales de salud graduados fueron enfermeros, médicos, farmacéuticos, fisioterapeutas, nutricionistas y cirujanos dentistas, entre otros, que mostraron las características teórico-metodológicas y los resultados de su formación profesional. Con base en el TAE, el período de recolección y análisis de datos se extendió de agosto de 2023 a agosto de 2024, después de finalizado el curso de especialización, y fue producto de una tesis doctoral en enfermería, teniendo como fuente de datos 337 Proyectos de Intervención (PI).

Los PI fueron organizados por un equipo de investigadores formados. Los documentos se introdujeron en *el programa informático* AtlasTi®. Los textos fueron codificados a la luz de la Teoría del Aprendizaje Experiencial, teniendo en cuenta a) caracterización sociodemográfica de los profesionales de la salud y posterior categorización según género, edad, formación, municipio de origen, centros de trabajo. b) tema del proyecto de intervención, dando lugar a las categorías: Diabetes Mellitus, Obesidad, Hipertensión Arterial Sistémica y Tabaquismo c) tipo de intervención propuesta, que dio lugar a las categorías: Educación en salud, Promoción y Gestión.

Los 337 proyectos de intervención se analizaron utilizando los principios de la teoría del aprendizaje experiencial de Kolb y siguieron una estrategia de saturación teórica. Durante el proceso de codificación y categorización de los datos, se analizaron continuamente nuevos temas hasta que no surgió ninguna información nueva que ampliara las categorías existentes. Este procedimiento garantizó que los datos analizados fueran suficientes para cumplir los objetivos del estudio y reflejar la variedad de intervenciones realizadas.

La información relacionada con la caracterización de los graduados se sometió a un análisis descriptivo simple, mientras que el resto se sometió a un preanálisis del contenido y exploración del material, seguido de la codificación y categorización de los datos, lo que condujo al tratamiento de los resultados, que incluyó la inferencia e interpretación teniendo en cuenta las recomendaciones de Prodanov y Freitas^(9)^ para la investigación cualitativa^(10)^.

El estudio se ajusta a las recomendaciones del *Consolidated Criteria for Reporting Qualitative Research* (COREQ). La investigación fue sometida y aprobada por el Comité de Ética bajo el número CAAE 66635322.0.0000.0121, número de dictamen 6.078.402. Se realizó de acuerdo con la Resolución 466 de 2012 del Consejo Nacional de Salud y la Ley General de Protección de Datos (LGPD), que establece las normas para el tratamiento de datos personales. Los autores declaran que no existen conflictos de interés en la realización de esta investigación. Los 337 IP fueron codificados para preservar el anonimato de los autores y etiquetados secuencialmente del IP 1 al IP 337.

## RESULTADOS

La caracterización de los formados reveló un predominio de mujeres (84,87%), siendo los enfermeros la mayoría (58,16%), seguidos de nutricionistas (13,65%), farmacéuticos (7,12%), cirujanos dentistas (4,45%), médicos (4,15%), fisioterapeutas (3,86%) y otros profesionales sanitarios (8,61%) (Tabla 1). La distribución de los profesionales por centros mostró que la capital, Florianópolis, tenía la mayor representación con 8 licenciados (24,92%), seguida de Blumenau con 69 (20,47%), Joinville con 61 (18,10%), Criciúma con 54 (16,04%), Chapecó con 48 (14,24%) y Lages con 21 (6,23%). Los temas de intervención se organizaron en los cuatro dominios de la Teoría del Aprendizaje Experiencial: Identificación, Análisis, Problematización y Transformación. Las afecciones más frecuentemente abordadas fueron la Diabetes Mellitus (36,40%), la Hipertensión Arterial Sistémica (22,90%), la Obesidad (15,80%) y el Tabaquismo (9,10%). Las intervenciones más realizadas fueron la educación en salud (37,43%), la promoción de la salud (32,30%) y la gestión sanitaria (15,21%). Florianópolis destacó como el centro con mayor número de intervenciones sobre Diabetes Mellitus (45%) e Hipertensión (32%).

**Tabla 1:** Distribución de los graduados por sexo, profesión y temas de intervención – Florianópolis, SC, Brasil, 2024. (n = 337)

Variables	Frecuencia (%)	Polos n (%)					
**Temas principales**		**Blumenau**	**Chapecó**	**Criciúma**	**Florianópolis**	**Joinville**	**Lages**
Diabetes Mellitus Hipertensión Arterial Obesidad Otros* Tabaquismo	164 (36,40) 103 (22,90) 71 (15,80) 71 (15,80) 41 (9,10)	26 (39,4) 15 (22,7) 9 (13,6) 11 (16,7) 5 (7,6)	28 (42,4) 13 (19,7) 10 (15,2) 10 (15,2) 5 (7,6)	25 (41) 12 (19,7) 11 (18) 7 (11,5) 6 (9,8)	45 (33,3) 32 (23,7) 19 (14,1) 25 (18,5) 14 (10,4)	30 (34,5) 20 (23) 17 (19,5) 14 (16,1) 6 (6,9)	10 (28,6) 11 (31,4) 5 (14,3)
**Intervenciones principales**		**Blumenau**	**Chapecó**	**Criciúma**	**Florianópolis**	**Joinville**	**Lages**
Educación en salud Promoción de salud Gestiónde salud Intervenciones tecnológicas Diagnóstico situacional Otros**	241 (37,43) 208 (32,30) 98 (15,21) 48 (7,45) 46 (7,14) 3 (0,47)	31 (33) 26 (27,7) 22 (23,4) 9 (9,6) 6 (6,4) 0	36 (38,3) 29 (30,9) 11 (11,7) 7 (7,4) 10 (10,6) 1 (1,1)	35 (36,5) 30 (31,3) 17 (17,7) 4 (4,2) 9 (9,4) 1 (1,1)	69 (34,7) 69 (34,7) 29 (14,6) 14 (7) 18 (9) 0	49 (43,4) 37 (32,7) 16 (14,2) 9 (8) 2 (1,8) 0	21 (43,8) 17 (35,4) 3 (6,3) 5 (10,4) 1 (2,1) 1 (2,1)

* Afecciones renales, mentales, neurológicas, respiratorias, cardiovasculares, endocrinas y neoplasias; tratamiento.

** Proceso de trabajo, identidad profesional, farmacia SUS.

En cuanto a las intervenciones realizadas por los profesionales, el 37,43% se centraron en la educación en salud, especialmente dirigidas a personas con Diabetes Mellitus e Hipertensión, con una fuerte participación de enfermeros y nutricionistas. Las intervenciones dirigidas a la promoción de la salud supusieron el 32,30%, con mayor participación de fisioterapeutas y farmacéuticos, especialmente en iniciativas de deshabituación tabáquica y control de la obesidad. Las intervenciones relacionadas con la gestión de la salud representaron el 15,21% de los proyectos, en los que enfermeros, médicos y dentistas desempeñaron un papel crucial en la planificación y el seguimiento de las intervenciones.

Cabe destacar el carácter interprofesional del 31% de las intervenciones, en las que participaron equipos formados por distintos profesionales sanitarios (enfermeros, nutricionistas, médicos, fisioterapeutas, entre otros), con el objetivo de lograr una atención más integrada y eficaz. Estas intervenciones interprofesionales fueron especialmente notables en la promoción y educación para la salud, donde la colaboración entre diferentes categorías profesionales dio lugar a enfoques multidisciplinares para gestionar enfermedades crónicas como la obesidad y el tabaquismo.

Por centros, las intervenciones más realizadas en Florianópolis se centraron en los temas de Diabetes Mellitus (45%) e Hipertensión (32%), con una fuerte presencia de enfermeros y médicos. Blumenau destacó en salud, especialmente en el control de la obesidad, con mayor participación de nutricionistas y fisioterapeutas. Joinville mostró una distribución equilibrada entre educación y promoción de la salud, con un papel importante de los farmacéuticos en las iniciativas para dejar de fumar (10%).

En Criciúma y Chapecó, la mayoría de las intervenciones se centraron en la educación en salud, con predominio de enfermeros y nutricionistas en las actividades de concienciación sobre la hipertensión y la diabetes. En Lages, en cambio, donde la representación de profesionales fue menor (6,23%), las acciones se dirigieron más al manejo de condiciones crónicas, con cirujanos dentistas y fisioterapeutas liderando programas de vigilancia de la salud bucal y actividades físicas para el control de la hipertensión.

## DISCUSIÓN

El Curso de Especialización en Atención a Personas con ECNT, ofrecido por la UFSC, ha contribuido significativamente para la calificación de los profesionales, destacándose la capilarización del curso en diversas regiones del estado de Santa Catarina. El predominio de mujeres entre los graduados refleja la realidad de la fuerza de trabajo en salud, con las mujeres desempeñando un papel clave en el cuidado de las ECNT. Este protagonismo es especialmente notable entre las enfermeras, que lideran la consolidación de la Atención Primaria de Salud (APS), identificando las necesidades de la comunidad y capacitando a las personas para cuidar de sus condiciones de salud^(11–13)^. Históricamente, la enfermería ha sido una profesión mayoritariamente femenina, mientras que áreas como la medicina, con mayor prestigio social, eran ocupadas por hombres^(11)^. Los profesionales de enfermería muestran una fuerte alineación con el TAE, utilizando su práctica diaria para resolver problemas y reflexionar sobre el conocimiento aplicado, reforzando su papel como líderes en la atención a las ECNT^(14)^.

La distribución de los graduados en los centros de Florianópolis, Lages, Blumenau, Joinville, Chapecó y Criciúma refuerza el alcance del curso. La diabetes (36,40%), seguida de la hipertensión (22,90%), la obesidad (15,80%) y el tabaquismo (9,10%) fueron las afecciones más prevalentes abordadas por los proyectos de intervención, lo que refleja la necesidad urgente de estrategias educativas y de promoción de la salud. Entre 2006 y 2020, la tendencia de la obesidad aumentó un 9,7%, pasando del 11,8% en 2006 al 21,5% en 2020. Durante el período analizado, la prevalencia de hipertensión y diabetes mellitus entre los adultos residentes en las capitales de los estados brasileños mostró una relativa estabilidad, con valores en torno al 25,2% y al 8,2%, respectivamente.La prevalencia de diabetes e hipertensión es mayor entre las personas con menor escolaridad, mientras que las que tienen más escolaridad muestran comportamientos más saludables^(15)^. Otra encuesta confirma que la proporción de personas con obesidad seguirá aumentando entre 2022 y 2023, pasando del 21,7% al 22,8%^(16)^.

El análisis de los proyectos reveló un amplio programa de formación, que capacita a los profesionales para abordar las enfermedades crónicas en diferentes territorios. El plan de ECNT está alineado con los indicadores y objetivos nacionales, tiene el potencial de impactar significativamente en la reducción de la morbilidad y mortalidad por ECNT y fortalecer los procesos de trabajo intersectorial y en red, promoviendo la innovación y nuevas competencias profesionales para la promoción de la salud^(17)^. Los datos refuerzan la tendencia mundial de aumento de las afecciones crónicas no transmisibles, que requieren acciones urgentes de prevención y control. En 2019, el 54,7% de las muertes en Brasil se atribuyeron a ECNT, en particular enfermedades cardiovasculares, cánceres, diabetes y enfermedades respiratorias crónicas. Estas enfermedades están estrechamente relacionadas con las condiciones de vida y el acceso desigual a los bienes y servicios públicos, como la educación, el empleo y la salud^(18)^.

El estado de Santa Catarina registró un aumento de sus tasas de mortalidad prematura por ECNT en el periodo estudiado y no alcanzó los objetivos fijados para el periodo^(19)^. La cobertura poblacional de los equipos de APS disminuyó entre 2017 y 2018 en el estado, y esto no se asoció con un aumento en la tasa de mortalidad prematura por ECNT. A pesar de la caída de las tasas de mortalidad prematura por ECNT en Brasil y de los esfuerzos en Atención Primaria, el control de esta mortalidad sigue siendo inestable^(19)^. Además de las políticas de prevención y control de las ENT, es fundamental analizar los factores que influyen en esta mortalidad prematura^(19)^.

Los temas tratados por los graduados fueron muy variados, abarcando áreas como la salud mental, las neoplasias, el estilo de vida, la gestión sanitaria, la atención integrada, las terapias alternativas, la salud específica de la población, la gestión e intervenciones farmacológicas, los aspectos profesionales y la identidad. Estos temas ponen de manifiesto la amplitud del curso y la complejidad de las ECNT. Los marcadores de morbilidad crónica, el autocuidado, los factores de riesgo y los perfiles de los usuarios, junto con la adherencia a los servicios y el enfoque multidisciplinar, son estrategias clave para la formación y la planificación en salud^(20)^.

Las intervenciones implementadas por los graduados, basadas en la Teoría del Aprendizaje Experiencial^(10)^, incluyeron educación en salud, promoción de la salud, manejo de condiciones crónicas y abordajes multidisciplinarios. Estas intervenciones no sólo se alinean con los principios de la teoría basada en un ciclo continuo de cuatro etapas (experiencia concreta (EC) – Actuar; observación reflexiva (OR) –Reflexionar; conceptualización abstracta (CA) – Conceptualizar; experimentación activa (EA) – Aplicar^(8)^), sino que también satisfacen las necesidades específicas de los usuarios, proporcionando un enfoque integrado y colaborativo para el cuidado de las condiciones crónicas. La educación en salud ha demostrado ser una herramienta transformadora, especialmente en temas como la diabetes, la hipertensión, la obesidad y el tabaquismo, proporcionando información práctica para el autocuidado y el cambio de comportamiento^(21,22)^. Las actividades comunitarias, como los paseos y los grupos de apoyo para dejar de fumar, refuerzan la importancia de la promoción de la salud para crear entornos que fomenten estilos de vida saludables^(22)^.

Las intervenciones basadas en la educación sanitaria son esenciales para que las personas gestionen mejor sus enfermedades crónicas. Los programas de educación sobre la diabetes, la hipertensión, la obesidad y el tabaquismo tienen como objetivo proporcionar información práctica y pertinente para que los usuarios comprendan mejor su enfermedad y adopten conductas de autocuidado. Estas intervenciones reflejan el ciclo de aprendizaje basado en la experiencia^(15,23)^, en el que se utilizan experiencias y pensamientos específicos para promover cambios duraderos en el comportamiento^(10,23)^.

Las actividades comunitarias que promueven estilos de vida saludables, como los paseos, las zonas de ocio y los grupos de alimentación sana, son ejemplos de estrategias que fomentan la salud pública. Estas actividades proporcionan oportunidades para la actividad física y también crean el entorno de apoyo necesario para el cambio de comportamiento. La promoción de la salud implica estrategias que consideran tanto los aspectos individuales, promoviendo estilos de vida más saludables, como los colectivos, reconociendo las condiciones que facilitan o dificultan la adopción de estas opciones^(22)^. Desde una perspectiva positiva, se centra en la salud y no en la enfermedad. La comprensión del enfoque salutogénico ha impulsado la innovación en la teoría y el diseño de intervenciones en salud que diferencian las acciones con un enfoque médico y de sistemas de atención^(24)^.

El cuidado de las enfermedades crónicas requiere un enfoque individualizado, que exige un plan de tratamiento individualizado que responda a las necesidades específicas de cada usuario, con intervenciones como el control periódico de la tensión arterial, el control de la dieta, el control de la glucemia y el control de la obesidad.La teoría de Kolb^(11,15)^ destaca la importancia de adaptar las estrategias de aprendizaje a las necesidades individuales, lo que se refleja en el desarrollo y la aplicación de estos programas. Cada interacción con un paciente es una oportunidad para aprender más sobre sus necesidades y ajustar los planes de tratamiento en consecuencia, promoviendo un ciclo continuo de aprendizaje y desarrollo, como los Planes Individualizados de Tratamiento. La TAE establece el proceso de aprendizaje para que los directivos, los equipos y las organizaciones resuelvan problemas, tomen decisiones, busquen oportunidades empresariales y formulen estrategias^(18)^. De este modo, se suele orientar a los directivos para que inicien programas que promuevan el «Aprendizaje Experiencial» y encuentren supuestos para su trabajo actual^(11,18,23)^.

La gestión de las enfermedades crónicas requiere un enfoque individualizado y continuo, centrado en intervenciones como la monitorización de la tensión arterial, el control de la glucemia y la nutrición. La TAE hace hincapié en la adaptación de las estrategias de aprendizaje a las necesidades de los pacientes, promoviendo el desarrollo de planes de tratamiento individualizados^(22)^. La interprofesionalidad surge como un factor esencial en la atención a los ECNT, siendo fundamental la colaboración de diversos profesionales de la salud, como nutricionistas, fisioterapeutas, psicólogos y médicos, para la implementación de estrategias más completas y eficaces^(22)^.

La formación ofrecida por la UFSC, que integró varias profesiones sanitarias, sirvió como ejemplo práctico de la aplicación del aprendizaje experiencial para desarrollar competencias interprofesionales esenciales para la gestión de la NCNC. Este enfoque facilitó la comprensión y el respeto de las funciones de cada profesional dentro del equipo, promoviendo una atención más coordinada e integrada^(15,22)^.

Para justificar las intervenciones basadas en la TAE, realizadas por profesionales de la salud capacitados y centradas en las enfermedades crónicas, es esencial comprender cómo estas acciones están en consonancia con los principios de esta teoría. Según Kolb, el aprendizaje tiene lugar a través de la transformación de la experiencia, en un proceso cíclico que combina experiencias concretas, reflexiones, abstracciones y experimentación^(8)^. Este enfoque se revela particularmente eficaz en la gestión de las enfermedades crónicas, ya que permite a los profesionales de la salud ajustar sus estrategias a las necesidades específicas de los usuarios, promoviendo cambios significativos en las prácticas asistenciales.

Los graduados del Curso de Especialización, a su vez, desarrollaron proyectos en seis centros de Santa Catarina, categorizados en cuatro grandes tipos de intervenciones: Educación en salud, Promoción de la salud y Gestión de enfermedades crónicas. Cada una de estas iniciativas se benefició del EAT, integrando conocimientos teóricos y práctica reflexiva. De esta forma, los profesionales pudieron no sólo abordar los factores de riesgo asociados a las condiciones crónicas – como se evidencia en estudios nacionales que reflejan el aumento de la obesidad y la prevalencia de hipertensión y diabetes tipo 2^(11,12)^ – sino también elevar la calidad de la atención y promover acciones que atiendan a las peculiaridades regionales.

Este enfoque ha permitido identificar áreas relevantes para la investigación, desarrollar directrices públicas más eficaces y aplicar acciones que respondan a las necesidades locales. De esta forma, la TAE proporcionó un marco teórico sólido para la formación continua de profesionales y para la mejora de la salud pública en Santa Catarina.

El uso de la Teoría del Aprendizaje Experiencial de Kolb^(15,23)^ permitió un abordaje práctico y reflexivo para los profesionales involucrados. La educación continuada, basada en experiencias reales y significativas, promovió la transformación de las prácticas de atención y gestión, destacando la importancia de la formación continuada y del aprendizaje significativo para la gestión de lasECNT^(25,26)^. Este enfoque está en consonancia con el Plan Nacional de Lucha contra las ENT 2021–2030, que destaca la importancia de la formación continuada para prevenir los factores de riesgo y promover la salud de la población^(18)^. La verificación de los datos en salud es esencial para tomar decisiones basadas en la evidencia, mejorar la gestión del sistema sanitario e implicar a las personas atendidas^(27)^.

La formación de los trabajadores de los distintos grupos profesionales favorece la actuación integral y cooperativa en la atención a las enfermedades crónicas. La teoría de Kolb^(11,15)^ destaca la importancia del aprendizaje basado en la experiencia en la colaboración interdisciplinar, donde el intercambio de conocimientos y experiencias entre especialistas promueve una gestión más eficaz de las enfermedades crónicas. Este tipo de interacción facilita la comprensión de la complejidad de cada afección y la creación de estrategias de tratamiento más completas y adecuadas para los pacientes, lo que refuerza la importancia de un enfoque multidisciplinar e integrado.

La educación en salud interprofesional fortalece la lógica colaborativa del trabajo en salud, buscando reducir la duplicación de actos profesionales, mejorar la comunicación, aumentar la satisfacción del usuario y garantizar la seguridad del paciente a través de la integración de acciones^(22)^. La interprofesionalidad promueve el trabajo colaborativo entre los profesionales de la salud, fortaleciendo las relaciones entre universidades, servicios y comunidades, además de desarrollar profesionales críticos y atender mejor las necesidades de la comunidad^(22)^.

La participación interprofesional y la práctica colaborativa son esenciales para una salud pública de calidad y para contribuir a la atención primaria de salud. Especialmente dada la creciente prevalencia de las enfermedades no transmisibles, las competencias básicas del trabajo profesional en equipo son esenciales para preparar a los trabajadores sociales y en salud del siglo^(28,29)^.

Los resultados de esta investigación subrayan la importancia de incorporar prácticas interprofesionales y formación continuada en la gestión de las enfermedades crónicas no transmisibles. La aplicación de la TAE de Kolb proporcionó una base práctica y reflexiva que facilitó la evolución de las prácticas asistenciales y de gestión de los participantes. La interprofesionalidad, que es el foco de las intervenciones examinadas, emerge como una estrategia importante para mejorar la calidad de la atención y hacer frente a las complejidades de la gestión de las ECNT, teniendo en cuenta los aspectos socioeconómicos y culturales.

El diferencial de las intervenciones basadas en la TAE radica en su carácter experiencial, que permite a los profesionales aplicar los conocimientos teóricos en contextos prácticos, reflexionando sobre los resultados para optimizar futuros abordajes. Este ciclo de aprendizaje continuo resultó especialmente eficaz para desarrollar competencias críticas y adaptar las estrategias a las necesidades locales y a las de otras regiones cubiertas por el curso.

El trabajo en equipo interprofesional es cada vez más necesario para la prestación eficaz de servicios de salud pública en entornos de atención primaria, en consonancia con los principios del SUS. Por lo tanto, las competencias interprofesionales deberían incorporarse a todos los programas de educación en salud y trabajo social. La innovación educativa en el desarrollo de las clínicas dirigidas por estudiantes ofrece una oportunidad única para evaluar y desarrollar dichas competencias^(30)^. Por último, destaca como factor clave la capilarización de la formación, con la distribución de los profesionales formados en diferentes regiones de Santa Catarina, lo que permite adaptar las intervenciones a las necesidades locales.

El aprendizaje experiencial, al valorizar la experiencia directa y la reflexión crítica, garantiza que los profesionales estén preparados para enfrentar los desafíos regionales y promover una salud pública más equitativa y eficaz^(8,22,23)^. Cabe destacar también el mérito de evaluar frecuentemente el clima de trabajo en equipo en los servicios de salud, con el objetivo de proporcionar subsidios para promover acciones dirigidas a mejorar este aspecto del trabajo interprofesional y, consecuentemente, promover un ambiente de trabajo más favorable a la colaboración interprofesional.

### Limitaciones e Indicaciones para Futuros Estudios

Aunque los datos se han explorado ampliamente, una posible limitación es que no se utilizaron todas las herramientas disponibles en el *software*AtlasTI®. Futuros estudios podrían explorar la adaptación de la Teoría del Aprendizaje Experiencial en otros estados brasileños.

## CONCLUSIÓN

Los proyectos de intervención desarrollados a lo largo del Curso de Especialización en Atención a Personas con Enfermedades Crónicas No Transmisibles han tenido un impacto significativo, contribuyendo a la formación y práctica interprofesional y ampliando las competencias en el manejo de las enfermedades crónicas. La aplicación de la Teoría del Aprendizaje Experiencial se destacó como estrategia para integrar la práctica reflexiva al contexto real, promoviendo transformaciones en los enfoques de atención y gestión. Las intervenciones realizadas abordaron condiciones como diabetes, hipertensión, obesidad y tabaquismo, en consonancia con las necesidades epidemiológicas locales y reforzando el papel de la educación continua en la construcción de soluciones adaptadas a las demandas regionales. El carácter interprofesional de las intervenciones, así como la promoción de la colaboración entre diferentes categorías profesionales, mostraron potencial para mejorar la coordinación de la atención y aumentar la calidad de vida de las comunidades atendidas.

Estos resultados indican que la combinación de formación especializada, aprendizaje experiencial y estrategias interprofesionales tiene el potencial de contribuir al fortalecimiento de la gestión de la atención de las enfermedades crónicas no transmisibles, alineando las prácticas de salud con las demandas sociales y epidemiológicas contemporáneas. El estudio refuerza la relevancia de modelos educativos que integren teoría y práctica como pilares para una formación continua y significativa, con impacto directo en la calidad de la atención a la salud.
